# Supplementary material for: Evolution and Dynamic Transcriptome of Key Genes of Photoperiodic Flowering Pathway in Water Spinach (Ipomoea aquatica)
Source: Int J Mol Sci. 2024 Jan 24;25(3):1420. doi: 10.3390/ijms25031420 (PMC10855745; doi:10.3390/ijms25031420)
Supplement: Supplementary file 1 [file ijms-25-01420-s001.zip › ijms-2785391-supplementary.pdf]

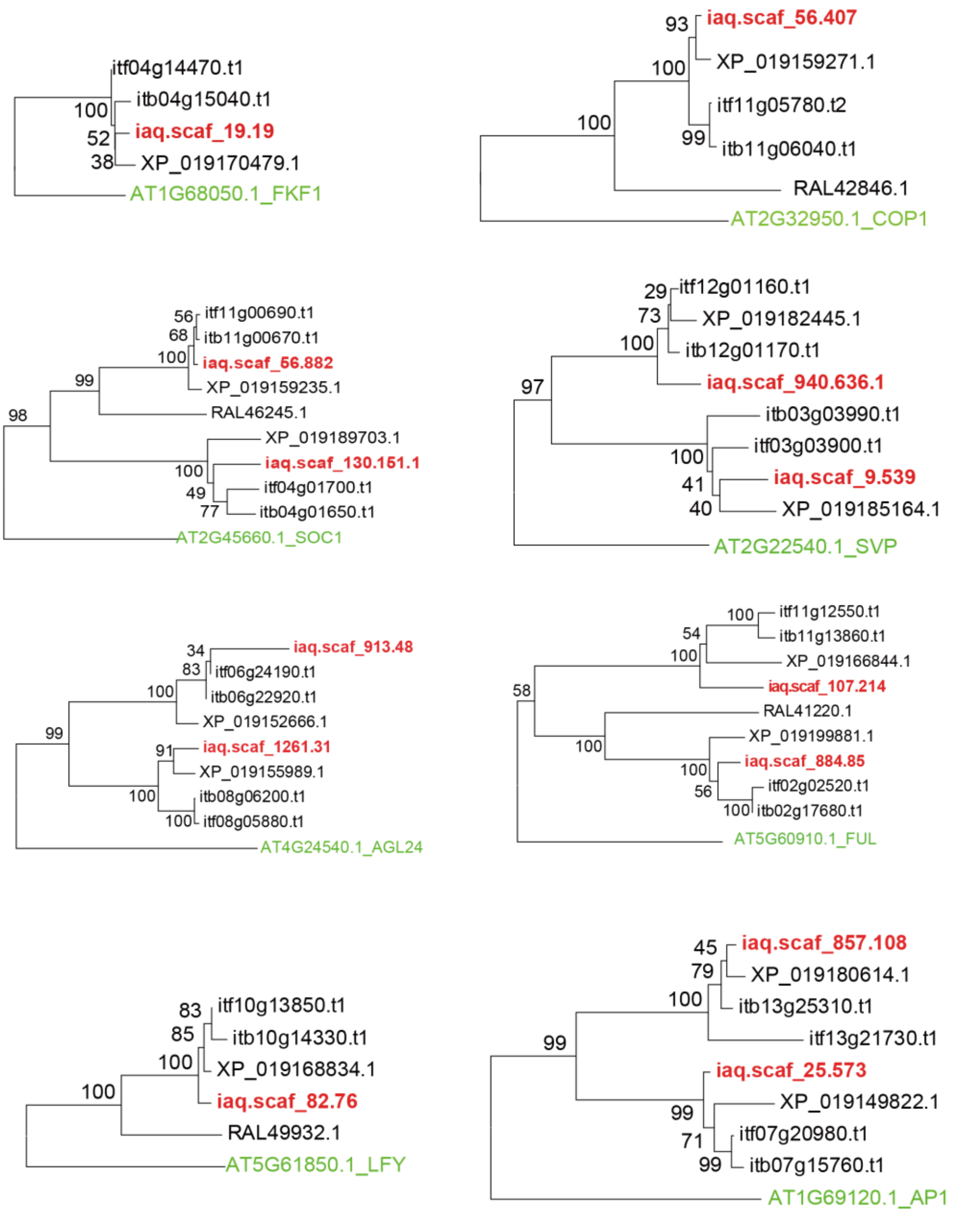

Figure S1. Phylogenetic analysis of different gene family in water spinach.

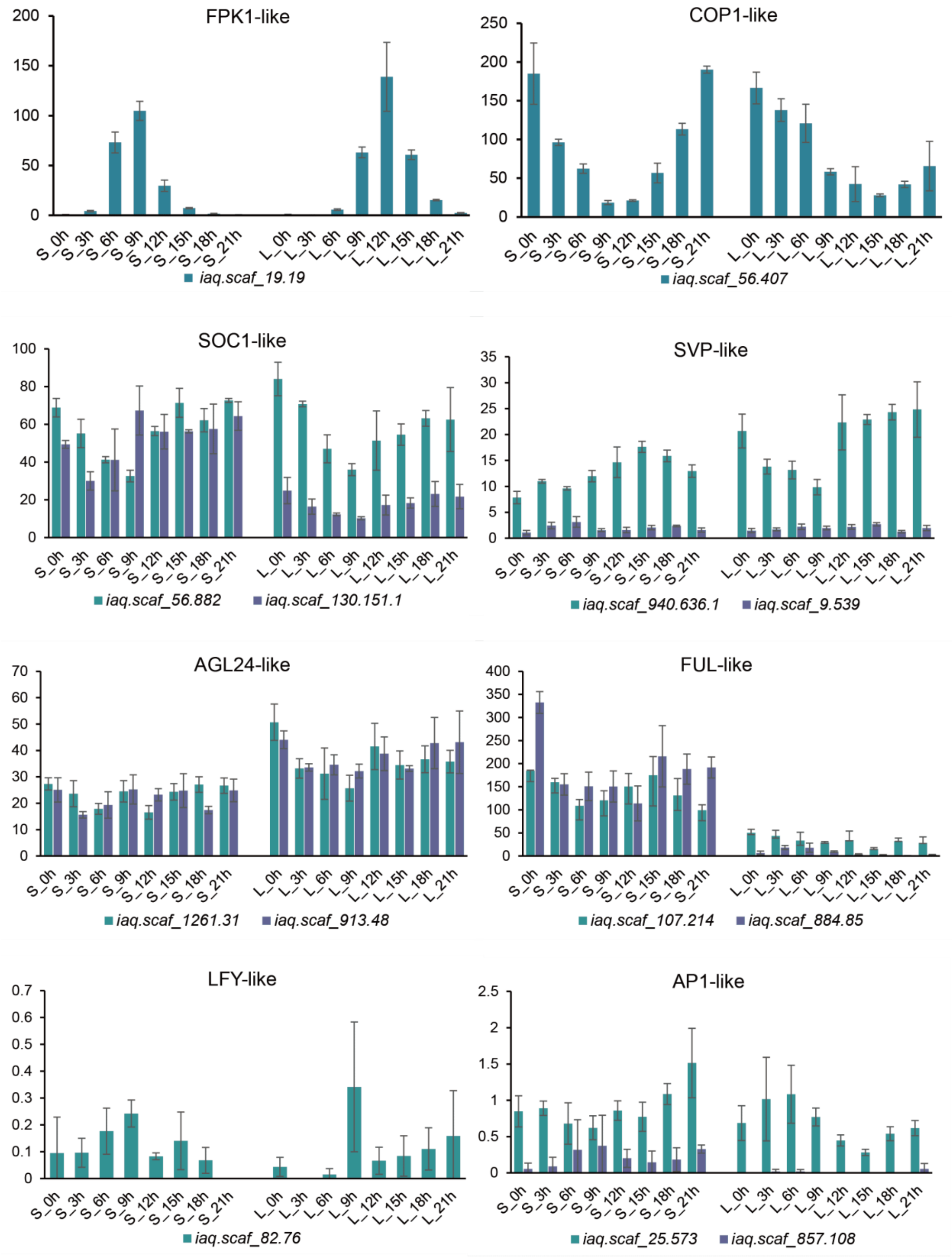

Figure S2. The transcript level of flowering-related genes in water spinach. S indicated short day; L indicated long day.

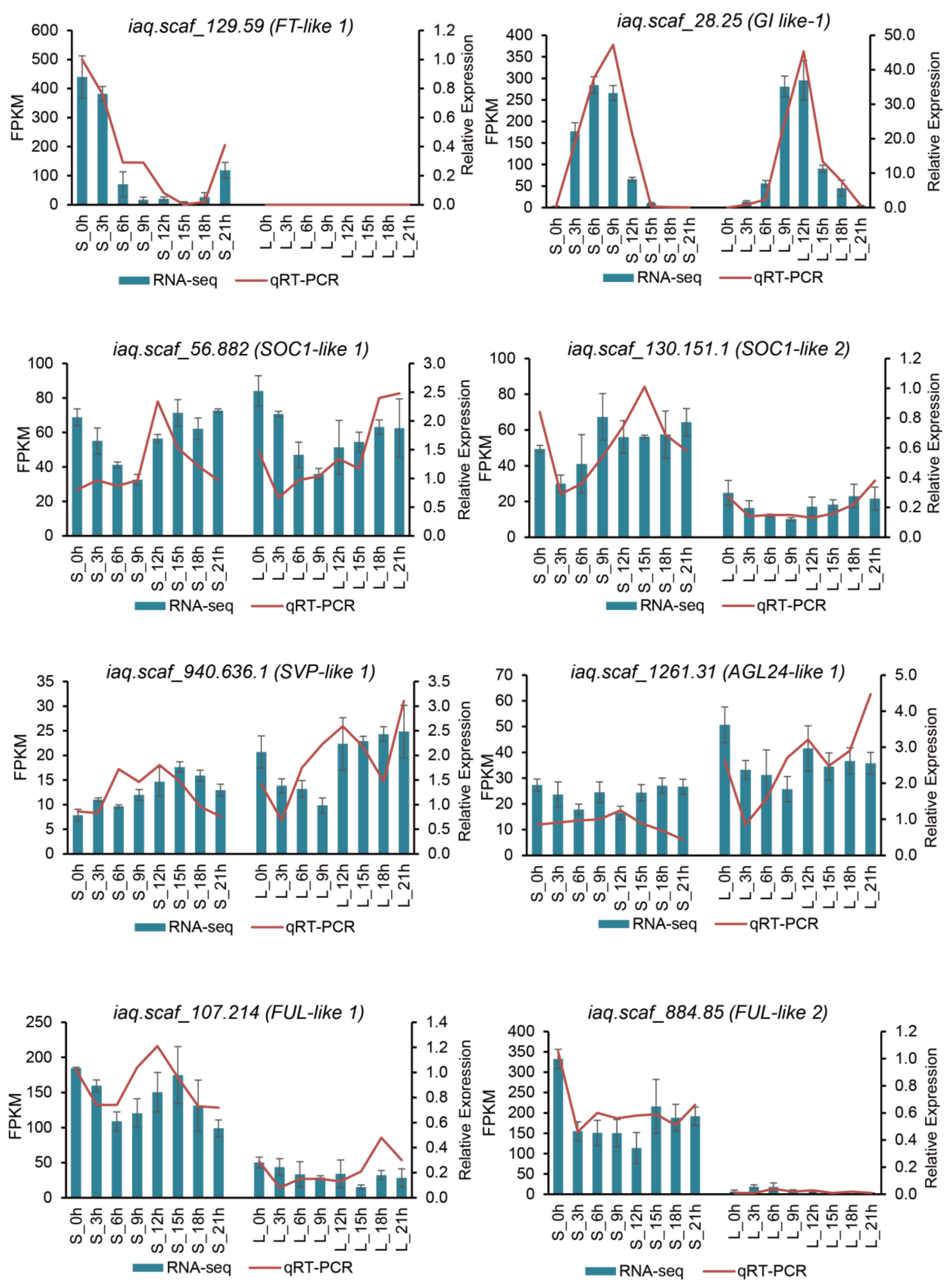

Figure S3. Comparative analysis of expression results between RNA-Seq and qRT–PCR for 8 flowering-related genes. Error bars represent standard deviation. S indicated short day; L indicated long day.

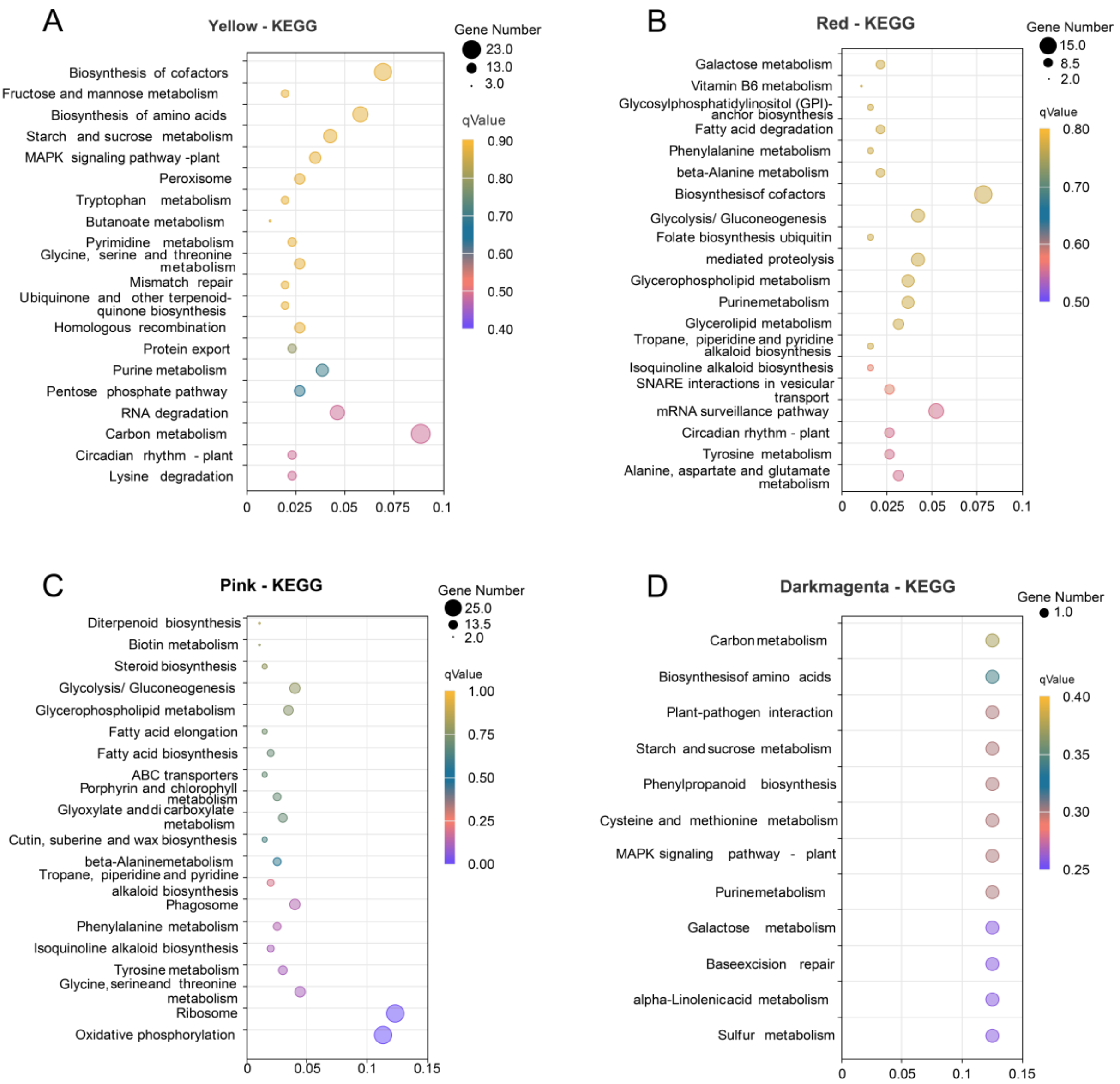

Figure S4. KEGG enrichment analysis in the four modules. A: yellow module; B: red module; C: pink module; D: darkmagenta module. . S indicated short day; L indicated long day.

Supplementary Table S1: The primer information for qRT-PCR

Gene ID	Primer name	Primer sequence (5'-3')	Product size (bp)
iaq.scaf_17.368(GAPDH)	GAPDH-F	CACTGTCCGAACTGAGAA	136
	GAPDH-R	GCAATCACCAACGAAGTC	
iaq.scaf_129.1643_iaq.scaf_129.1644(CCA1)	CCA1-F	TTGTTGAAGCCTTGAAGTTGT	142
	CCA1-R	CCATCACCATCATTGCCC	
iaq.scaf_51.23(LHY)	LHY-F	ACTCTATGGGCGGGCTTG	181
	LHY-R	CGGTTTCCTCTTTGGTCGT	
iaq.scaf_884.166.1(TOC1)	TOC1-F	TCTCGCAACAAAGGATGGA	118
	TOC1-R	CTTGGAATGTAATACGGTGGAT	
iaq.scaf_9.675(CO_1)	CO_1-F	AAGACGACGAAGACGAAGC	181
	CO_1-R	GGGACAAAGTAATGGTGCG	
iaq.scaf_129.59(FT_1)	FT_1-F	CAGGAATGGGTATGAGATGAGG	129
	FT_1-R	GGAGAGGCTGGGTTTGGA	
iaq.scaf_28.25(GI_1)	GI_1-F	GAACAATGGTCCACTCGCT	164
	GI_1-R	TCCACTGACTTCACTAACTGCC	
iaq.scaf_1261.31(AGL24_1)	AGL24_1-F	GCTCGCCCTCATCGTCTT	177
	AGL24_1-R	ATTTCCTTGCTCAGTCTCACAT	
iaq.scaf_56.882(SOC1_1)	SOC1_1-F	GTCTGTGCTGTGTGATGCTGA	158
	SOC1_1-R	CCTGTTCTTGTTCCACTGATG	
iaq.scaf_130.151.1(SOC1_2)	SOC1_2-F	GCGACAAGCAGACAGGTGA	111
	SOC1_2-R	GGGAGAGAAGATGATGAGAGC	
iaq.scaf_107.214(FUL_1)	FUL_1-F	AGAAAAAGGACAGAGCATTGAA	140
	FUL_1-R	TGGAACATAGGAAGATGAGGAG	
iaq.scaf_884.85(FUL_2)	FUL_2-F	CCTCCTATGTTGTGCCGC	101
	FUL_2-R	CTGACGCTGAGTTCCTTCTG	
iaq.scaf_940.636.1(SVP_1)	SVP_1-F	GTCTGTGCTGTGCGATGC	105
	SVP_1-R	TTCCGTCTTTCAAGTATTCCC	
